# Cutaneous Cancer Trends in Spain: An Emerging Epidemic with Shifting Tumor Types

**DOI:** 10.3390/jcm14165654

**Published:** 2025-08-10

**Authors:** Jorge Santos-Juanes, Raquel Santos-Juanes, Marta López-Pando, Marta García-Puente, Miguel Plaza-López, Celia Gómez de Castro, Laura Palacios-García, Cristina Galache

**Affiliations:** 1 GRIDER, Grupo de Investigación en Dermatología, Universidad de Oviedo; 33006 Oviedo, Spain; santosjjorge@uniovi.es (J.S.-J.); celiagomez_88@hotmail.com (C.G.d.C.); cristinagalache@gmail.com (C.G.); 2 Unidad de Gestión Clínica de Dermatología, Hospital Universitario Central de Asturias, 33006 Oviedo, Spain; martalopezpando@gmail.com (M.L.-P.); martagpuente@gmail.com (M.G.-P.); miguel.plaza@sespa.es (M.P.-L.); laura.palacios@sespa.es (L.P.-G.); 3 Área de Dermatología, Departamento de Medicina, Facultad de Medicina, Universidad de Oviedo, Avda Julián Clavería s/n, 33006 Oviedo, Spain; 4 Instituto de Investigación Sanitaria del Principado de Asturias (ISPA), 33011 Oviedo, Spain; 5 Instituto Universitario de Oncología del Principado de Asturias, University of Oviedo, 33006 Oviedo, Spain

**Keywords:** skin cancer, melanoma, basal cell carcinoma, squamous cell carcinoma, incidence

## Abstract

**Background/Objectives**: The incidence of both non-melanoma and melanoma skin cancers has increased significantly in recent years. Understanding their epidemiology is essential for implementing effective community prevention strategies and optimizing healthcare resource planning. This study aimed to (1) quantify the number of skin tumors diagnosed at the Central University Hospital of Asturias (HUCA) from 2014 to 2023; (2) describe temporal trends in the incidence of basal cell carcinoma (BCC), squamous cell carcinoma (SCC), and melanoma; and (3) analyze the clinical and histopathological features of melanoma and estimate age-standardized incidence rates. **Methods**: A retrospective study was conducted using data from the Pathology Registry of HUCA. Diagnosed cases were collected in an Excel database and analyzed with SPSS v.27. Quantitative variables were expressed as mean ± standard deviation; categorical variables as counts and percentages. Incidence rates and 95% confidence intervals were calculated using EPIDAT 4.2, based on the European standard population. Municipal population data were obtained from the SADEI website. **Results**: A total of 7477 BCC, 2689 SCC, and 585 melanoma cases were identified. Over the study period, BCC cases increased by 50%, SCC by 80%, and melanoma by 159%. Among melanoma cases, 89% were diagnosed by dermatologists, 60% were women, 33.1% were melanoma in situ, and 73.3% were superficial spreading melanomas. Age-standardized melanoma incidence rose from 8.85/100,000 in 2014 to 18.50 in 2023. **Conclusions**: Skin cancer incidence, especially melanoma, has risen markedly in Asturias, with more in situ cases suggesting improved early detection. The 2020 decline reflects COVID-19′s impact on access to care, underscoring the need for sustained prevention and screening efforts.

## 1. Introduction

Melanoma and non-melanoma skin cancer (NMSC) represent the most frequently diagnosed malignant neoplasms among individuals of Caucasian descent [1]. NMSC encompasses primarily basal cell carcinoma (BCC) and squamous cell carcinoma (SCC) [2]. In fair-skinned populations, it is estimated that 90–95% of skin cancers are attributable to ultraviolet (UV) radiation, highlighting the predominant role of environmental exposure in their pathogenesis [3]. This etiological profile suggests that a significant proportion of these malignancies could be prevented through behavioral changes and effective UV protection strategies.

Globally, the incidence of both melanoma and NMSC continues to rise, largely driven by increased exposure to UV radiation. BCC is strongly associated with episodes of intense, intermittent UV exposure during childhood and adolescence [4], whereas SCC is more commonly linked to chronic, cumulative UV exposure over several decades [5]. Melanoma risk appears to be influenced by both intermittent and sustained UV radiation exposure patterns [6].

### 1.1. Basal Cell Carcinoma

BCC is the most common malignancy in humans, accounting for approximately 70–80% of all non-melanoma skin cancers. It typically arises on sun-exposed areas of the skin and rarely involves mucosal surfaces or acral sites such as the palms and soles. BCC is generally characterized by slow growth and a low metastatic potential. Although it is rarely life-threatening, delayed diagnosis or inadequate treatment can lead to significant local tissue destruction and disfigurement [7].

Clinically, BCC most commonly presents as a flesh-colored or pink pearly papule, often accompanied by surface ulceration or telangiectatic vessels. The majority of lesions are located on the head and neck, although the trunk and extremities may also be affected [8].

Management of BCC includes various surgical modalities, such as standard excision, electrodesiccation and curettage, cryosurgery, and Mohs micrographic surgery. These techniques are primarily employed for localized disease and are associated with excellent outcomes, achieving five-year cure rates exceeding 95%.

### 1.2. Squamous Cell Carcinoma

SCC is the second most common malignant cutaneous neoplasm, accounting for approximately 20–30% of NMSCs [9]. It predominantly occurs on chronically sun-exposed skin in elderly individuals with fair skin. Clinically, SCC often presents as an exophytic, keratotic lesion with a propensity for ulceration. As the tumor evolves, it may enlarge progressively, become indurated, and adhere to underlying structures. Although distant metastasis is relatively rare, regional lymph nodes are the most frequent site of dissemination.

Surgical excision with histologically confirmed complete tumor removal remains the treatment of choice for SCC. When appropriately managed, cure rates exceed 90%. In advanced or inoperable cases, systemic oncologic therapies and/or radiotherapy may be necessary [10].

### 1.3. Melanoma

Melanoma is a malignant neoplasm arising from the malignant transformation of melanocytes, which are derived from neural crest cells. While the vast majority of melanomas originate in the skin, they may also develop in extracutaneous locations where neural crest cells migrate, including the brain, gastrointestinal tract, and eye [11].

The four major subtypes of primary cutaneous melanoma are as follows [12]:

Superficial spreading melanoma (SSM): The most common subtype, accounting for approximately 70% of cases. It can occur on any cutaneous site, but most frequently affects the lower extremities and trunk. Clinically, it presents as a flat lesion with focal elevation and exhibits the classical ABCD features of melanoma: asymmetry, border irregularity, color variegation (light and dark brown, black, blue, red, or gray), and a diameter greater than 6 mm. It is characterized by a prolonged radial growth phase and delayed vertical invasion.

Nodular melanoma (NM): Comprises 15–20% of primary melanomas, yet it is responsible for up to 40% of melanoma-related deaths. It may arise on any skin site, most commonly the head, neck, and trunk. Clinically, it appears as a thickened plaque or a dome-shaped, polypoid, or exophytic lesion. Pigmentation varies widely and may include blue, black, or amelanotic presentations. It demonstrates rapid vertical growth without a preceding radial phase, often resulting in substantial Breslow thickness at diagnosis.

Acral lentiginous melanoma (ALM): Represents 2–5% of all melanomas and predominantly affects older individuals. It is located on acral surfaces, including the palms, soles, and subungual regions. Clinically, it presents as a pigmented macule with interspersed papules or nodules in hues of brown, black, blue, or depigmented areas. It has a slow growth pattern.

Lentigo maligna melanoma (LMM): Accounts for 5–10% of melanomas, typically occurring in elderly patients with chronically sun-damaged skin. Commonly affected areas include the face, neck, and dorsal hands. Clinically, it manifests as a flat pigmented macule with focal elevation, often exhibiting blue and gray tones. It evolves from a precursor in situ lesion (lentigo maligna (LM)) that may grow slowly over decades and is characterized by an irregularly bordered brown-to-black pigmentation.

Although melanoma has traditionally been considered a relatively low-prevalence malignancy, its incidence has steadily increased over the past five decades, particularly among fair-skinned populations of European ancestry. This rising trend is largely attributed to increased exposure to ultraviolet (UV) radiation—both solar and artificial—which is a well-established etiological factor in melanoma pathogenesis. Recent estimates indicate that more than 75% of melanoma cases are attributable to UV radiation exposure [13].

Breslow thickness remains the most powerful prognostic indicator, followed by patient age and ulceration status. It is defined as the depth of tumor invasion from the top of the epidermal granular layer to the deepest malignant melanocyte. In ulcerated tumors, measurement is taken from the base of the ulcer [14].

Surgical excision remains the cornerstone of melanoma treatment. For invasive melanomas with a Breslow thickness > 2 mm, a clinical excision margin of 20 mm is typically recommended. In situ melanomas are generally managed with 5–10 mm margins or with margin-controlled techniques such as Mohs micrographic surgery.

The eighth edition of the American Joint Committee on Cancer (AJCC), implemented in 2018, represents the current standard for melanoma staging [15].

The true incidence of non-melanoma skin cancer is often underestimated, as it is not consistently included in national cancer registries and is frequently limited to the first tumor diagnosed in each patient [16].

This study aimed to (1) quantify the number of skin tumors diagnosed at the Central University Hospital of Asturias (HUCA) from 2014 to 2023; (2) describe temporal trends in the incidence of basal cell carcinoma (BCC), squamous cell carcinoma (SCC), and melanoma; and (3) analyze the clinical and histopathological features of melanoma and estimate age-standardized incidence rates.

## 2. Materials and Methods

An observational, retrospective, and descriptive study was conducted, including all cases diagnosed between 1 January 2014 and 31 December 2023. The study was approved by the Research Ethics Committee of the Principality of Asturias (Study number: 2024-433).

Cases were identified through the computerized database of the Department of Pathology (PatWin^®^). Systematic searches were performed using diagnostic codes for BCC, SCC, cutaneous melanoma, and skin. For BCC and SCC, annual tumor counts were recorded.

For melanoma, the following variables were collected:

Medical record number; year of biopsy; age at diagnosis (in years); sex: 0 = male; 1 = female; histological subtype: 1 = superficial spreading melanoma; 2 = lentigo maligna melanoma; 3 = nodular melanoma; 4 = acral lentiginous melanoma; 5 = other types; anatomical location: 1 = trunk; 2 = head; 3 = extremities; 4 = acral; 5 = genital; specialty that performed the initial biopsy or excision: 1 = dermatology; 2 = plastic surgery; 3 = maxillofacial surgery; 4 = primary care; 5 = otorhinolaryngology; 6 = general surgery; Breslow thickness (in millimeters), with a value of 0 for melanoma in situ cases.

Each case was individually verified by reviewing the electronic medical record (Millennium^®^) to confirm the patient’s residence. Only patients residing in Health Areas II and IV of the Asturias Health Service were included. Non-cutaneous tumors were excluded. Tumor staging was established based on data extracted from the medical record.

### 2.1. Quality Control

Data collection for melanoma followed the quality criteria proposed by Lomas (Supplementary Table S1) [16].

### 2.2. Statistical Analysis

Statistical analysis was performed using IBM SPSS Statistics version 27.0 (IBM Corp., Armonk, NY, USA). Melanoma qualitative variables were expressed as absolute frequencies and percentages. The chi-square (χ^2^) test was used for comparison of proportions.

### 2.3. Incidence Rates and Trend Analysis

Annual crude incidence rates per 100,000 inhabitants were calculated. For age-standardized melanoma incidence rates, the direct method was used, with the European standard population as reference. This operation was performed using EPIDAT version 4.2 (Xunta de Galicia, Santiago de Compostela, Spain), based on municipal census data published by the Asturian Society of Economic and Industrial Studies (SADEI).

Temporal trends in incidence rates were analyzed by estimating the Annual Average Percent Change (AAPC), applying log-linear regression models to the annual rates. This analysis enables estimation of the mean annual percentage change in the incidence of each type of skin cancer.

Generative artificial intelligence (GenAI, Chat GPT 4.0, OpenAI, Inc., San Francisco, CA, USA) has been used in this paper to generate graphics.

## 3. Results

### 3.1. Skin Tumors

Between 2014 and 2023, a total of 10,751 skin tumors were diagnosed at the Central University Hospital of Asturias (HUCA, Oviedo), of which 7477 (69.54%) were basal cell carcinomas (BCC), 2689 (25.01%) were squamous cell carcinomas (SCC), and 585 (5.44%) were melanomas (Table 1).

The average annual crude incidence rates were 208.97 per 100,000 inhabitants for BCC, 75.15 per 100,000 for SCC, and 16.35 per 100,000 for melanoma. As shown in the table, the number of basal cell carcinomas increased by 293 cases between 2014 and 2023, representing a cumulative increase of 47%. In the case of squamous cell carcinomas, the increase was 153 tumors, corresponding to an 80% rise. Melanoma cases increased by 73, which represents a 159% rise over the study period.

**Figure 1:**
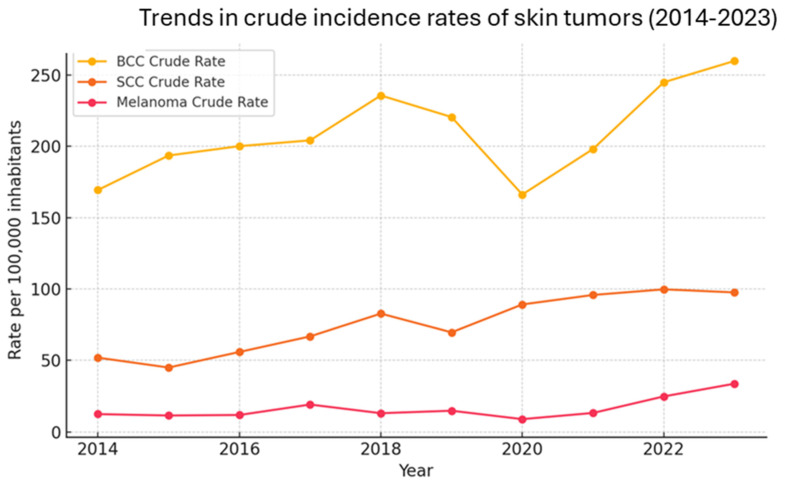
Trends in crude incidence rates of skin tumors.

The annual crude incidence rates (per 100,000 inhabitants) for each tumor type, along with trend lines. SCC demonstrates a strong and consistent upward trend (R^2^ = 0.87), while BCC and melanoma display moderate annual increases with more variability between years (R^2^ = 0.35) (Figure 1). The estimated average annual percent increase was +2.93% for BCC, 9.27% for SCC, and 8.02% for melanoma.

**Figure 2:**
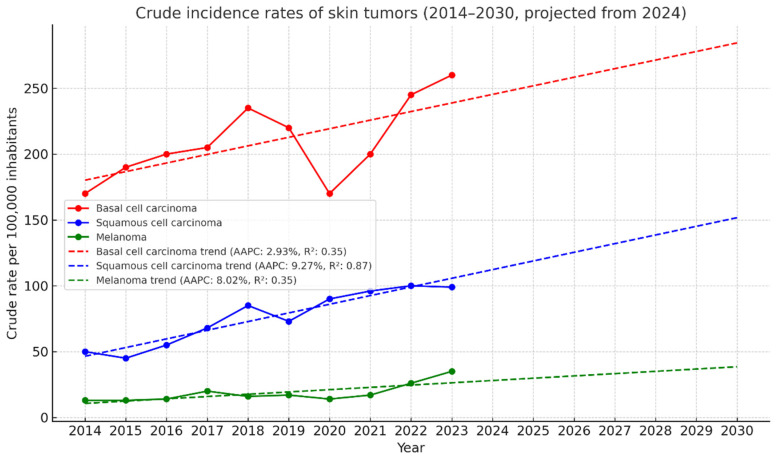
Projection of crude incidence rates of skin tumor through 2030.

Overall, Figure 2 highlights an upward trend in the incidence of all three types of skin cancer. BCC consistently showed the highest incidence rates throughout the study period, with a sustained growth trajectory projecting to reach 300 cases per 100,000 inhabitants by 2030. The smoothed curve indicates a clear acceleration starting in 2020, possibly reflecting improved diagnostic access following the COVID-19 pandemic. SCC shows a more gradual but steady increase, with projected rates approaching 120 cases per 100,000 by the end of the projection period. This trend may be associated with cumulative factors such as population aging and chronic sun exposure. Although melanoma exhibited lower absolute incidence rates, it displayed a continuous and progressive upward trend. The smoothed projection suggests melanoma will surpass 40 cases per 100,000 inhabitants by 2030, underscoring the importance of maintaining secondary prevention efforts (Figure 2).

### 3.2. Melanoma

#### 3.2.1. Clinical Variables

A total of 585 new cases of cutaneous melanoma were recorded over the 10-year study period (2014–2023), affecting 576 patients. Of these, 60% were female. The mean age at diagnosis was 64.8 ± 15.79 years, with the youngest patient being 19 years old and the oldest 100 years.

#### 3.2.2. Annual Distribution of Melanoma by Sex

Table 2 shows a consistent predominance of female cases throughout the study period, comprising 60% of all melanomas diagnosed, compared to 40% in males. The number of cases in males increased by 33 between 2014 and 2023 (+157%), while in females, the increase was 40 tumors (+160%), leading to an overall increase of 159%.

#### 3.2.3. Anatomical Location and Sex

Regarding tumor location, the most commonly affected site was the extremities (*n* = 224; 38.3%), followed by the trunk (*n* = 196; 33.5%), head and neck (*n* = 113; 19.3%), acral areas (*n* = 51; 8.7%), and genital area (*n* = 1; 0.2%).

Table 3 presents the distribution of melanoma cases by anatomical site and sex (*p* < 0.001). In men, the predominant site was the trunk (44.9%), followed by the head and neck (25.6%) and extremities (20.1%). Acral and genital involvement was uncommon (9.0% and 0.4%, respectively). In contrast, in women, the extremities were the most frequent site (50.4%), followed by the trunk (25.9%) and head/neck (15.1%).

#### 3.2.4. Histological Subtypes and Sex

The most frequent histological subtype was superficial spreading melanoma (*n* = 423; 72.3%), followed by lentigo maligna (*n* = 58; 9.9%), nodular melanoma (*n* = 54; 9.2%), acral lentiginous melanoma (*n* = 43; 7.4%), and other variants (*n* = 7; 1.2%). The median Breslow thickness was 1 mm (range: 0.4–2.5). The “other” category includes six desmoplastic melanomas and one spitzoid melanoma.

As shown in Table 4, no statistically significant differences in histological subtype were observed between sexes (*p* = 0.179).

#### 3.2.5. In Situ and Invasive Melanoma by Year

In Table 5, the distribution of melanoma by sex and tumor type (in situ vs. invasive) is shown. No significant differences were observed between sexes in terms of melanoma type (in situ vs. invasive) (*p* = 1.000).

**Figure 3:**
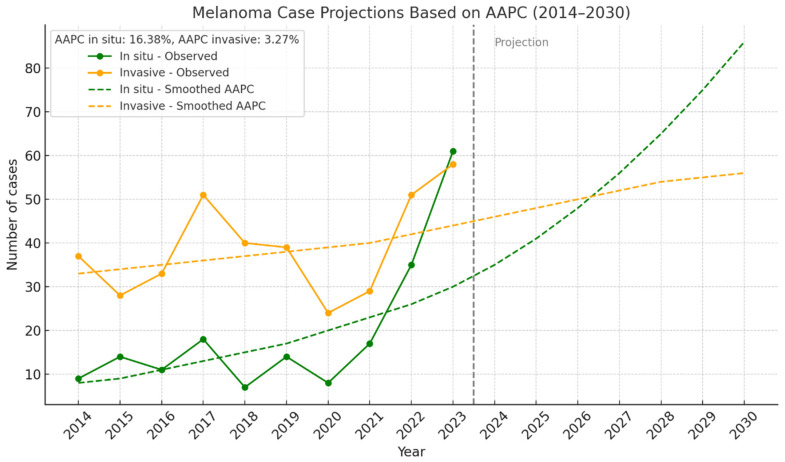
Projection of melanoma cases based on AAPC (2014–2030).

Figure 3 illustrates the smoothed trend (Annual Average Percent Change, AAPC) from 2014 to 2023: in situ melanoma showed a marked annual increase (AAPC: +16.38%, R^2^ = 0.451), suggesting improved early detection. Invasive melanoma displayed a milder increase (AAPC: +3.27%, R^2^ = 0.114), indicating higher data variability or other influencing factors. A projection based on the AAPC, indicating a sustained increase in in situ melanoma diagnoses, potentially doubling in a few years if the trend continues.

#### 3.2.6. Melanoma by Breslow Thickness Categories

**Figure 4:**
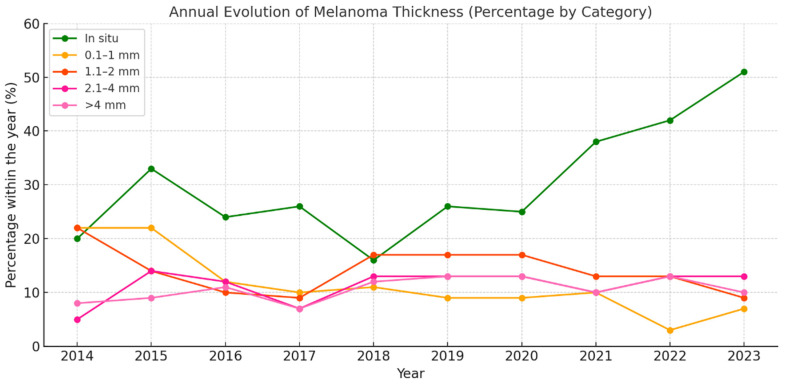
Annual evolution of melanoma thickness (percentage by category).

Figure 4 illustrates the annual evolution of the different melanoma thickness subgroups, revealing a rising trend in the diagnosis of in situ melanomas, which accounted for 51.3% of all cases in 2023. This category, highlighted in green in the graph, shows a sustained increase, particularly since 2020, suggesting an enhancement in early detection. This improvement may be attributed to advances in dermatological care, screening campaigns, or increased public awareness.

In contrast, invasive categories have tended to remain stable or exhibit a slight relative decline. Specifically, melanomas measuring 0.1–1 mm, which represent thin but already invasive lesions, have shown a progressive decrease since 2017. The thicker categories (>2 mm) have remained relatively constant at around 10–13% of total cases, indicating that although early diagnosis is improving, a significant proportion of melanomas are still detected at an advanced stage.

Overall, this evolution points to a shift toward earlier-stage diagnosis, which translates into an improved overall prognosis and reflects the positive impact of early detection strategies implemented over the past decade.

#### 3.2.7. Melanoma by Clinical Stage

**Figure 5:**
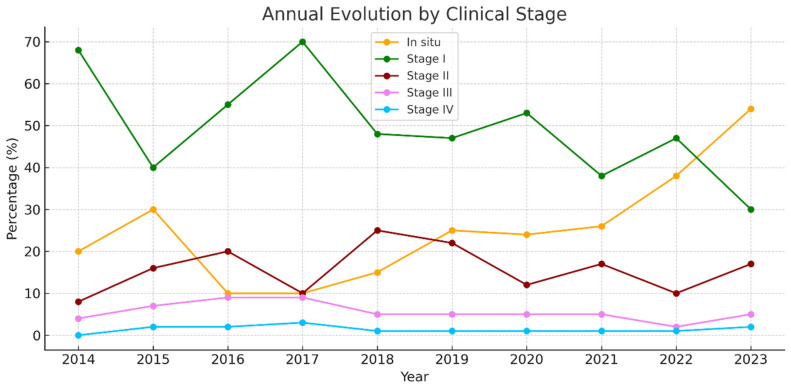
Annual evolution by clinical stage.

Figure 5 shows that 89.3% of cases were either in situ or stage I (≤2 mm). In parallel, the proportion of melanomas diagnosed at more advanced stages (stages II, III, and IV) has either decreased or remained stable. Notably, stages III and IV have been infrequent in recent years, indicating a positive impact on reducing the number of cases with poor prognosis at the time of diagnosis.

Overall, the data reflect a favorable shift toward early-stage detection, which has direct implications for prognosis, treatment, and healthcare burden. Nearly 80% of cases are in situ melanomas or stage I.

#### 3.2.8. Specialist Responsible for the Diagnosis

**Figure 6:**
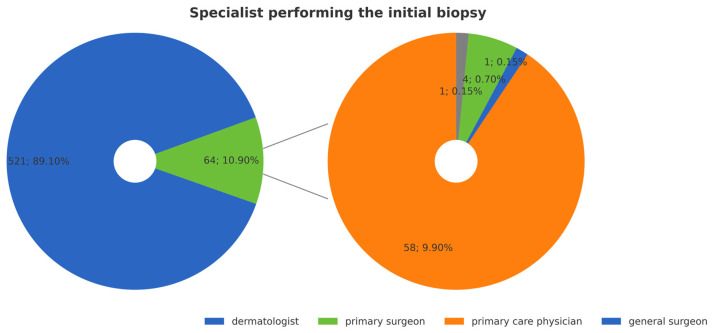
Specialist performing the initial biopsy.

Dermatologists were responsible in 89% of cases (Figure 6).

#### 3.2.9. Crude and Age-Standardized Incidence Rates

The lowest rate was in 2020 (6.56 per 100,000), possibly reflecting underdiagnosis during the COVID-19 pandemic. From 2021 onwards, a sustained rise led to the highest rate in 2023 (18.5 per 100,000) (Table 6).

## 4. Discussion

Skin cancer is the most common type of cancer worldwide, and its incidence continues to rise [10]. Although numerous epidemiological studies have been conducted, most have focused on non-Spanish populations [17,18,19,20,21]. Despite its high prevalence, data on the incidence of skin cancer in Spain remain limited, underscoring the importance of studies such as the present one.

Between 2014 and 2023, a total of 10,751 skin tumors were diagnosed at the Central University Hospital of Asturias (HUCA): 7477 (69.54%) BCC, 2689 (25.01%) SCC, and 585 (5.44%) melanomas. Understanding the local epidemiology of these tumors is essential for designing effective prevention campaigns and for planning appropriate healthcare resources. It is noteworthy that the global market for skin cancer treatment was valued at USD 7.2 billion in 2021 and is projected to reach USD 14.5 billion by 2030 [22]. According to our projections, SCC will continue to increase rapidly, exceeding 180 cases per 100,000 population by 2030. Melanoma would almost double its rate compared to 2023 if current trends persist, while BCC is expected to show a more gradual increase.

### 4.1. Basal Cell Carcinoma

In most studies, BCC accounts for approximately 80% of NMSCs, and SCC for about 20% [1,23]. However, some reports describe a more balanced distribution (50–50%) [24]. In our series, the proportion of BCC decreased from 78% in 2014 to 72% in 2023, while SCC increased from 22% to 28%, suggesting a relative increase in SCC incidence compared to BCC.

A study conducted in Valencia reported a mean annual increase in BCC incidence of 3.91%, significantly higher in women (8.28% annually) than in men (0.92% annually). The lifetime risk of developing BCC was estimated at 5.8% overall, 5.02% in women, and 7% in men [25].

Over the ten-year study period, BCC incidence in our series showed a progressive increase. Depending on the methodology applied, this increase was estimated at approximately 3% annually, or a 47% relative increase from the first to the last year of the study. These findings align with international studies reporting a 5% annual increase [26] and with Spanish studies showing a 4% annual rise [25].

### 4.2. Squamous Cell Carcinoma

Globally, SCC incidence is increasing at an approximate rate of 7% annually [27], which is consistent with the 8% annual rise observed in our study. The crude incidence rate found was 97.6 SCCs per 100,000 population, significantly higher than the 38.6 per 100,000 reported in a systematic review conducted in Spain [2]. These differences are likely due to the earlier timeframe of that meta-analysis (1998–2013), which predates the current study period.

The increase in NMSC incidence has been partially attributed to the aging population, as age is a major risk factor [28].

In 2020, coinciding with the COVID-19 pandemic, there was a significant drop in BCC diagnoses, which was not observed for SCC. This difference may be attributed to clinical presentation: BCC typically presents as small, slow-growing lesions, whereas SCC often appears as large, ulcerated, and bleeding tumors that prompt earlier medical attention. These findings are consistent with those reported in a recent meta-analysis [29].

### 4.3. Cutaneous Melanoma

Regarding sex distribution, melanoma was more frequent in women than in men, a pattern similar to that described in European countries such as Denmark, the United Kingdom, and Norway. This contrasts with findings from the United States, Canada, Australia, and New Zealand, where incidence is higher among men. These differences may be due to varying sun exposure patterns, screening practices, and sociocultural and biological factors across regions [30].

In our series, the most commonly affected anatomical sites were the extremities (38.3%), followed by the trunk (33.5%). Among men, the trunk was the most frequent site (44.9%), followed by the head and neck (25.6%), while in women, the extremities were the most affected (50.4%), followed by the trunk (25.9%). These results are consistent with findings from previous studies in U.S. and Swedish populations, where melanoma in men most commonly affects the trunk, followed by the head and neck, and then the extremities; in contrast, extremities are the most affected sites in women, similar to our cohort [30,31].

In our series, the most frequent histological subtype was superficial spreading melanoma (72.3%), followed by lentigo maligna (9.9%), nodular melanoma (9.2%), acral lentiginous melanoma (7.4%), and other types (1.2%). These results align with the existing literature, which reports superficial spreading melanoma accounting for approximately 70% of all melanomas. We observed similar frequencies of lentigo maligna and acral lentiginous melanomas, while the proportion of nodular melanomas was lower than that reported in other series [12].

We observed an increase in melanoma incidence during the study period, from 8.85 cases per 100,000 population in 2014 to 34.04 cases in 2023. This trend may be related to cumulative sun exposure habits in the population now at higher risk and to improved diagnostic accuracy due to the widespread use of dermoscopy [32]. A notable decrease was observed in 2020 (6.56 cases per 100,000) compared to 2019 (9.2 per 100,000), coinciding with the onset of the COVID-19 pandemic. This decline may be attributed to reduced access to both primary and specialized care, a phenomenon reported nationwide [33].

The rising incidence trend has also been documented in other studies conducted in Spain [32,34,35,36], and the rates recorded in our series are among the highest in the country [32,34,37,38,39].

Importantly, our study demonstrated an increase in the diagnosis of early-stage melanomas over time, as evidenced by a higher proportion of in situ melanomas and tumors with Breslow thickness < 1 mm and early-stage disease. This suggests improved early detection, likely due to screening campaigns, heightened public awareness, and access to specialized dermatologic care. This trend has also been reported in a study conducted in Mallorca [32]. It is important to note that while diagnostic improvements and demographic aging likely contribute to rising melanoma and NMSC incidence, the absence of stratified or age–period–cohort modeling in our analysis limits our ability to differentiate these effects from generational shifts or changes in sun exposure behavior across birth cohorts [40].

The observed rise in early-stage melanoma diagnoses, particularly in situ lesions, underscores the need to strengthen public health interventions aimed at primary and secondary prevention. Targeted UV protection campaigns should be promoted, especially among younger individuals and high-risk groups, with emphasis on the dangers of intermittent sun exposure and the importance of photoprotection behaviors. Routine skin self-examinations and access to regular dermatological check-ups should be encouraged, particularly for individuals with fair skin, a personal or family history of melanoma, or occupational sun exposure. In addition, integrating skin cancer prevention messaging into primary care settings and school-based health education may contribute to earlier recognition. Finally, ensuring equitable access to dermatological care across both urban and rural areas is crucial to reducing potential disparities in early diagnosis.

This study has both strengths and limitations. Among its strengths is the inclusion of all histologically confirmed skin tumor cases, ensuring diagnostic validity.

Limitations include the following:

1. The lack of data on relevant confounding factors such as skin phototype, immunosuppression status, rural versus urban residence, socioeconomic level, and access to healthcare services. These variables are known to influence skin cancer risk and incidence trends, but they were not available in the pathology registry used for this analysis. Future studies incorporating these determinants are necessary to gain a more comprehensive understanding of the epidemiological patterns observed.
2. This was a cross-sectional study with no clinical follow-up, preventing assessment of outcomes or prognosis.
3. Melanomas diagnosed in the private healthcare sector were not included. The literature indicates that this exclusion may result in an underestimation of true incidence due to incomplete reporting of cases diagnosed in outpatient settings not captured by official epidemiological surveillance systems [13]. Underreporting of melanoma has also been documented in certain periods within the Surveillance, Epidemiology, and End Results (SEER) program [13].

## 5. Conclusions

Skin cancer incidence, particularly melanoma, has increased substantially in Asturias over the past decade. The growing proportion of in situ melanomas suggests improvements in early detection, likely driven by heightened public awareness and dermatological vigilance. A temporary decline in diagnoses during 2020 highlights the disruptive impact of the COVID-19 pandemic on cancer screening and access to care. These findings reinforce the importance of maintaining robust skin cancer prevention and early detection strategies.

## Figures and Tables

**Table 1:** The annual number of BCCs, SCCs, and melanomas over the study period, including the crude incidence rates for each tumor type and the overall number of skin cancers.

	2014	2015	2016	2017	2018	2019	2020	2021	2022	2023	Total
Population	367,844	364,233	361,319	359,500	357,745	356,436	355,586	352,868	349,953	352,493	3,577,977
BCC	623	705	723	734	843	786	591	699	857	916	7477
Crude incidence rate—BCC	169.37	193.56	200.10	204.17	235.64	220.52	166.20	198.10	244.89	259.86	208.97
SCC	191	164	202	240	296	248	317	338	349	344	2689
Crude incidence rate—SCC	51.92	45.03	55.91	66.76	82.74	69.58	89.15	95.79	99.73	97.59	75.15
Melanoma	46	42	43	69	47	53	32	47	87	119	585
Crude incidence rate—melanoma	12.51	11.53	11.90	19.19	13.14	14.87	9.00	13.32	24.86	33.76	16.35
Total tumors	860	911	968	1043	1186	1087	940	1084	1293	1379	10,751

**Table 2:** Distribution of melanoma by sex.

Year	Men *n* (%)	Women *n* (%)	Total
2014	21 (45.7%)	25 (54.3%)	46
2015	15 (35.7%)	27 (64.3%)	42
2016	18 (41.9%)	25 (58.1%)	43
2017	29 (42.0%)	40 (58.0%)	69
2018	17 (36.2%)	30 (63.8%)	47
2019	23 (43.4%)	30 (56.6%)	53
2020	10 (31.2%)	22 (68.8%)	32
2021	10 (21.3%)	37 (78.7%)	47
2022	37 (42.5%)	50 (57.5%)	87
2023	54 (45.4%)	65 (54.6%)	119
Total	234 (40.0%)	351 (60.0%)	585

**Table 3:** Distribution of melanoma by anatomical site and sex (*p* < 0.001).

Gender	Trunk	Head/Neck	Extremities	Acral	Genital	Total
Men	105 (44.9%)	60 (25.6%)	47 (20.1%)	21 (9.0%)	1 (0.4%)	234
Women	91 (25.9%)	53 (15.1%)	177 (50.4%)	30 (8.5%)	0 (0.0%)	351
Total	196 (33.5%)	113 (19.3%)	224 (38.3%)	51 (8.7%)	1 (0.2%)	585

**Table 4:** Histological subtype and gender.

Gender	SSM	LM	NM	ALM	Other	Total
Men	160 (68.4%)	29 (12.4%)	23 (9.8%)	17 (7.3%)	5 (2.1%)	234 (100.0%)
Women	263 (74.9%)	29 (8.3%)	31 (8.8%)	26 (7.4%)	2 (0.6%)	351 (100.0%)
Total	423 (72.3%)	58 (9.9%)	54 (9.2%)	43 (7.4%)	7 (1.2%)	585 (100.0%)

**Table 5:** Melanoma by sex and tumor type.

Year	In Situ (*n*, %)	Invasive (*n*, %)	Total
2014	9 (19.6%)	37 (80.4%)	46
2015	14 (33.3%)	28 (66.7%)	42
2016	10 (23.3%)	33 (76.7%)	43
2017	18 (26.1%)	51 (73.9%)	69
2018	7 (14.9%)	40 (85.1%)	47
2019	14 (26.4%)	39 (73.6%)	53
2020	8 (25.0%)	24 (75.0%)	32
2021	18 (38.3%)	29 (61.7%)	47
2022	36 (41.4%)	51 (58.6%)	87
2023	61 (51.3%)	58 (48.7%)	119
Total	195 (33.3%)	390 (66.7%)	585

**Table 6:** Melanoma crude and age-standardized rates.

Year	Crude	Age-Standardized	CI (95%)
2014	12.5053	8.8519	6.3781–12.5397
2015	11.5311	7.7178	5.4370–11.2829
2016	11.9008	8.7172	6.2704–12.4742
2017	19.1933	12.3394	9.4036–16.5768
2018	13.1378	8.8098	6.2883–12.7656
2019	14.8694	9.2957	6.7200–13.2855
2020	8.9992	6.5627	4.3572–10.3103
2021	13.3194	8.6537	6.1995–12.6959
2022	24.8604	14.2388	11.1150–18.8906
2023	33.7595	18.4991	15.0767–23.4161

## Data Availability

The data presented in this study are available on request from the corresponding author (accurately indicate status).

## Supplementary Materials

The following supporting information can be downloaded at: https://www.mdpi.com/article/10.3390/jcm14165654/s1, Supplementary Table S1. Criteria modified from Loma’s.

## Abbreviations

The following abbreviations are used in this manuscript:

HUCA	Central University Hospital of Asturias HUCA
BCC	Basal cell carcinoma
SCC	Squamous cell carcinoma
NMS	Non-melanoma skin cancer
SSM	Superficial spreading melanoma
LMM	Lentigo maligna melanoma
LM	Lentigo maligna
NM	Nodular melanoma
ALM	Acral lentiginous melanoma
UV	Ultraviolet
TNM	Tumor, node, metastasis classification
AJCC	American Joint Committee on Cancer
