# Supplementary material for: Cutaneous Cancer Trends in Spain: An Emerging Epidemic with Shifting Tumor Types
Source: J Clin Med. 2025 Aug 10;14(16):5654. doi: 10.3390/jcm14165654 (PMC12387036; doi:10.3390/jcm14165654)
Supplement: Supplementary file 1 [file jcm-14-05654-s001.zip › jcm-3794646-supplementary.pdf]

Supplementary Table S1. Criteria modified from Loma's.

**Criterion**	**Description**
1. Data source	Department of Pathology registry
2. Counting method	Number of tumors and number of patients
3. Inclusion criteria	Primary cutaneous tumors
4. Histological verification	Histological confirmation in all cases
